# Coronary Endarterectomy: a Case Control Study and Evaluation of Early Patency Rate of Endarterectomized Arteries

**DOI:** 10.21470/1678-9741-2018-0402

**Published:** 2020

**Authors:** Mario Augusto Cray da Costa, André Luís Betero, Jefferson Okamoto, Marcelo Schafranski, Elise Souza dos Reis, Ricardo Zanetti Gomes

**Affiliations:** 1 Department of Medicine, Universidade Estadual de Ponta Grossa - Campus Uvaranas, Ponta Grossa, Paraná, Brazil.; 2 Clinica Cray da Costa Medicina e Pesquisa, Ponta Grossa, Paraná, Brazil.; 3 Universidade Estadual de Ponta Grossa, Campus Uvaranas, Ponta Grossa, Paraná, Brazil.

**Keywords:** Cardiopulmonary Bypass, Coronary Artery Bypass, Endarterectomy, Morbity, Myocardial Infarctation, Stroke, Length of Stay

## Abstract

**Objective:**

To compare two groups of patients - the coronary endarterectomy group, with patients undergoing coronary artery bypass grafting (CABG) with coronary endarterectomy (CE), and the control group, with patients undergoing CABG without CE. We analyzed the rate of major outcomes (perioperative acute myocardial infarction [AMI], stroke, and mortality) and minor outcomes (time of cardiopulmonary bypass [CPB], time of aortic clamp, and postoperative length of hospital stay). We also determined the rates of early graft patency in patients undergoing CE.

**Methods:**

We reviewed a database of patients submitted to CABG, with or without associated CE, between January 2011 and June 2017. Twenty-five patients submitted to CE were compared with 201 patients submitted only to conventional surgery; the two groups presented similar preoperative characteristics and all the European System for Cardiac Operative Risk Evaluation (EuroSCORE) II variables did not presented statistically significant difference. We considered statistically significant values of *P*< 0.05.

**Results:**

There was no statistically significant difference in relation to time of post-surgical hospitalization (*P*=0.8139), incidence of perioperative AMI (*P*=0.2976), stroke (*P*=0,2976), and mortality rate (*P*=1.0000), but endarterectomy was associated with longer aortic clamping time (*P*=0.0004) and CPB time (*P*=0.0030). The rate of patency evaluated in patients submitted to endarterectomy (78,95%) was compatible with that described in the literature.

**Conclusion:**

In this sample, coronary endarterectomy was associated with the rate of early graft patency similar to that of the literature, with morbidity and mortality rates similar to those of conventional surgery.

**Table:** 

Abbreviations, acronyms & symbols				
AMI	= Acute myocardial infarction		DM	= Diabetes mellitus
CABG	= Coronary artery bypass grafting		EuroSCORE	= European System for Cardiac Operative Risk Evaluation
CAD	= Coronary artery disease		LAD	= Left anterior descending
CCS	= Canadian Cardiovascular Society		LITA	= Left internal thoracic artery
CE	= Coronary endarterectomy		LV	= Left ventricle
CEG	= Coronary endarterectomy group		MGB	= Marginal branch
CG	= Control group		NYHA	= New York Heart Association
CPB	= Cardiopulmonary bypass		PDA	= Posterior descending artery
DAPT	= Dual antiplatelet therapy		RCA	= Right coronary artery
DGB	= Diagonal branch			
DGsB	= Diagonalis branch			

## INTRODUCTION

Surgical treatment is the standard procedure for patients with complex coronary artery disease (CAD), aiming at a complete myocardial revascularization^[1]^. In patients with diffuse CAD, characterized by long threads affected by atherosclerosis, coronary endarterectomy (CE) may be necessary, with the purpose of obtaining a complete revascularization or facilitating the anastomosis of seriously calcified arteries^[2]^. The factors that increase the likelihood of diffuse CAD include advanced age, prior percutaneous coronary intervention, and comorbidities, such as diabetes mellitus (DM) and dyslipidemias^[3-5]^.

CE was introduced in the 1950s, as an optional treatment of severe atherosclerosis and myocardial ischemia. Bailey et al. were the first to perform it, blindly using distal retrograde technique and without the use of cardiopulmonary bypass (CPB). It was performed in an isolated way, since coronary artery bypass grafting (CABG) would be developed only in the 1960s. In 1958, for the first time, Longmire et al. performed CE anterogradely, under direct vision (open endarterectomy)^[1,3,6-8]^.

Many surgeons have been reluctant to perform CE because initial studies showed high rates of mortality and morbidity, as well as acute myocardial infarction (AMI), so the procedure has been used only sporadically and with a highly selected group of patients^[1,3,6]^. Mortality by up to 30 days after the procedure for open or closed technique is usually similar. On the other hand, more than one CE would be associated with higher rates of mortality, as well as the use of the saphenous vein in the CABG procedure^[9]^.

Although the literature reports higher risk of CABG with CE than without it, CE has been demanded in order to provide complete revascularization, because of the increased incidence of diffuse or complex coronary heart disease in patients referred for surgery. On the other hand, advances in the surgical technique and perioperative management allowed better results after CE, especially with the use of dual antiplatelet therapy (DAPT)^[3,10]^.

The aim of this research is to compare two groups of patients: the coronary endarterectomy group (CEG), with patients undergoing CABG with CE, and the control group (CG), with patients undergoing CABG without CE. We analyzed the rate of major outcomes (perioperative AMI, stroke, and mortality) and minor outcomes (time of CPB, time of aortic clamp, and postoperative length of hospital stay). We also determined the rates of early graft patency in patients undergoing CE.

## METHODS

This is a retrospective, observational, and analytical case control study. We used the database of the cardiac surgery service of the Santa Casa de Misericórdia de Ponta Grossa, Paraná, Brazil. These data were collected prospectively and include European System for Cardiac Operative Risk Evaluation (EuroSCORE) II data, as well as other characteristics of the surgery (number and types of grafts, time of aortic clamp and of CPB, postoperative complications, etc.).

We analyzed the database and collected data from all patients with CAD undergoing CABG between January 2011 and June 2017 (n=697). We divided the patients into two groups: CEG was formed by patients undergoing CABG with incidental or programmed CE (n=25) and CG was formed by patients submitted to isolated CABG, without CE, using CPB (n=201).

The inclusion criteria were patients undergoing CABG requiring CPB and complete data on the datasheet. We excluded 471 patients with insufficient data, or when other surgical procedures were necessary in addition to myocardial revascularization, or patients submitted to CABG without CPB. And we compared the major outcomes (perioperative AMI, stroke rates, and mortality rates) and the minor outcomes (time of CPB, time of aortic clamp, and length of hospital stay after surgery).

The AMI criteria were c-troponin values > 10 times the 99^th^ percentile upper reference limit in addition to one of the following elements: (a) development of new pathological Q waves; (b) angiographic documented new graft occlusion or new native coronary artery occlusion; (c) imaging evidence of new loss of viable myocardium or new regional wall motion abnormality consistent with an ischaemic aetiology.

The stroke criterion was the presence of clinical symptoms of duration longer than one hour, compatible with ischemic injury, and associated to change in computed cranial tomography.

Patients undergoing CE were followed up prospectively and those who accepted it were submitted to angiography or coronary angiotomography in order to determine vessel patency rates, three months after surgery. We considered the following criteria to determine the patency rates: (I) absence of obstructive lesion, (II) obstructive lesion ≤ 50%, (III) obstructive lesion > 50%, and (IV) occlusion.

We tested continuous variables for normality. Variables with normal distribution were presented as “mean” and the not normal ones were presented as “median”. We performed the analysis of qualitative variables through Fisher’s exact test, we analyzed the quantitative variables through Student's *t*-test (normal distribution) or Mann-Whitney U test (not normal distribution), and we considered a statistically significant value of *P*<0.05. The analyses were carried out with the aid of the Medcalc® software, version 17.5.5 (MedCalc Software bvba, Belgium).

### Endarterectomy Technique

In this study, we performed CE using the following technique: expanded arteriotomy (though not the totality of the artery) with withdrawal of the plate by eversion, followed by irrigation of the treated artery flatbed, with solution containing 1 mL of heparin and 9 mL of 0.9% saline solution. Subsequently, still in the operating room or immediately after admission of the patient into the intensive care unit, we performed the passage of nasogastric tube and administered 300 mg of clopidogrel. We recommended the continuous use of DAPT. We preferably used pedicle left internal thoracic artery (LITA) for left anterior descending (LAD) artery and saphenous veins for other coronaries. We used the no-touch technique to dissect the saphenous vein and proximal anastomosis to the aorta before CPB in order to dilate the vein graft using aortic pressure, avoiding excessive pressure in the endothelium by manual dilation.

## RESULTS

Preoperative data, such as gender, age, DM, DM on insulin, kidney function, function of the left ventricle (LV), presence of class IV angina (Canadian Cardiovascular Society [CCS]), functional class of New York Heart Association (NYHA), and EuroSCORE II value, are listed in Table 1. There was no statistically significant difference, demonstrating the similarity of the two groups.

**Table 1:** Patients’ preoperative characteristics.

		CEG (n=25)	CG (n=221)	*P*-value
Male		19 (76%)	149 (74.1%)	10.000
Age, years (median)		60	64	0.1351
DM		13 (52%)	55 (32.7%)	0.0734
Use of insulin		5 (20%)	17 (10.1%)	0.1730
Creatinine clearance	> 85 ml/min	8 (40%)	79 (48.5%)	
	50-85 ml/min	11 (55%)	77 (47.2%)	0.5437
	< 50 ml/min	1 (5%)	6 (3.7%)	
	In dialysis	0 (0.0%)	1 (0.6%)	
NYHA Class	I	4 (20%)	20 (12.3%)	
	II	8 (40%)	52 (31.9%)	0.4197
	III	8 (40%)	80 (49.1%)	
	IV	0 (0.0%)	11 (6.7%)	
Ejection fraction	> 50%	22 (88%)	171 (85.1%)	
	31-50%	2 (8%)	21 (10.4%)	0.7446
	21-30%	1 (4%)	9 (4.5%)	
	≤ 20	0 (0.0%)	0 (0.0%)	
Angina class IV (CCS)		10 (40%)	81 (40.3%)	10.000
EuroSCORE II (median)		16.300	20.300	0.6745

CCS=Canadian Cardiovascular Society; CEG=coronary endarterectomy group; CG=control group; DM=diabetes mellitus; EuroSCORE=European System for Cardiac Operative Risk Evaluation; NYHA=New York Heart Association

Perioperative characteristics (urgency of the surgery, CPB time, aortic clamp time, average of grafts, postoperative AMI and stroke, hospital stay time after surgery, and mortality) are listed in Table 2. We performed CE in 8.4% of the bypassed coronary arteries. The average of aortic clamp time was higher in the CEG (65.6 minutes) than in the CG (51.5 minutes) (*P*=0.0004). The same occurred with CPB time, which had higher median in the CEG (95 minutes) than in the CG (73 minutes) (*P*=0.0030).

**Table 2:** Patients’ perioperative characteristics.

	CEG (n=25)	CG (n=221)	*P*-value
Urgency	4 (16.0%)	51 (25.4%)	0.4580
Aortic clamp time (mean)	65.6 min	51.5 min	0.0004
CPB time (median)	95.0 min	73.0 min	0.0030
Grafts	2.92 (± 1.41)	3.01 (± 0.70)	0.7160
Postoperative AMI	1 (4.0%)	2 (1.0%)	0.2976
Stroke	1 (4.0%)	2 (1.0%)	0.2976
Mean hospitalization time after surgery (days)	6	6	0.8139
Death	2 (8%)	23 (11.4%)	10.000

AMI=acute myocardial infarction; CEG=coronary endarterectomy group; CG=control group; CPB=cardiopulmonary bypass

The mortality in CEG was 8%, while in CG it was 11.4%, but there was no statistically significant difference. The same occurred with stroke and AMI, which presented no difference between the groups. Among CEG patients who died, two were submitted to LAD endarterectomy and one was submitted to endarterectomy of diagonal branch (DGB).

We performed CE in an incidental manner in 96.3% of vessels (n=26), and the purposeful way (scheduled) was the exception. The artery undergoing CE with greater frequency (50%) was the LAD artery (13 cases). The frequencies of other vessels are in Table 3.

**Table 3:** Frequency of vessels undergoing endarterectomy.

Coronary artery	N	%
RCA	6	22.22
LAD	13	48.15
DGB	3	11.11
DGsB	1	3.70
PDA	2	7.41
MGB	2	7.41
Total	27	100
	**N**	**%**
One vessel	23	92.0
Two vessels	2	8.0
Total	25	100

DGB=diagonal branch; DGsB=diagonalis branch; LAD=left anterior descending; MGB=marginal branch; PDA=posterior descending artery; RCA=right coronary artery

Nineteen CEG patients agreed to perform angiotomography or coronary angiography three months after surgery, and 78.95% (n=15) of the vessels submitted to CE were pervious. The data on the patency rates are presented in Tables 4 and 5.

**Table 4:** Graft patency in the arteries subjected to endarterectomy (N=19).

Patency rates/vase	LAD, N (%)	DGB	MGB	RCA	PDA	Total
No injury	1 (16.7%)	0 (0%)	1 (50%)	0 (0%)	1 (50.0%)	5 (26.3)
≤ 50% lesion	2 (33.3%)	2 (100%)	1 (50%)	2 (28.6%)	0 (0%)	7 (36.8)
> 50% lesion	3 (50.0%)	0 (0%)	0 (0%)	2 (28.6%)	0 (0%)	3 (15.8)
Occlusion	0 (0%)	0 (0%)	0 (0%)	3 (42.8%)	1 (50%)	4 (21.1)
Total	6 (31.6%)	2 (10.5%)	2 (10.5%)	7 (36.8%)	2 (10.5%)	19 (100%)

DGB=diagonal branch; LAD=left anterior descending; MGB=marginal branch; PDA=posterior descending artery; RCA=right coronary artery

**Table 5:** Comparison between graft patency in the arteries subjected to endarterectomy (N=19) by territory.

Territory/patency rates	Pervious	Occluded	*P*-value
Left coronary	10 (100%)	0 (0.00%)	0.0325
Right coronary	5 (33.33%)	4 (66.67%)	
Total	15 (78.95%)	4 (21.05%)	

## DISCUSSION

CE consists of removal of the atheromatous plaque, dissection of the vascular wall, and removal of the internal and middle layers involved in the atherosclerotic process, preserving the adventitia and restoring the lumen of the artery^[2]^. Therefore, CE is a surgical procedure associated with CABG surgery. It is usually performed in patients with severe CAD. It is accomplished through coronary arteriotomy and extraction of the atheromatous plaque that occludes the vessel, with posterior graft from the internal thoracic artery or saphenous vein, allowing irrigation of the ischemic tissue. The internal thoracic artery is preferably used due to better patency rates, lower thrombogenicity, and increased resistance to atherosclerosis than the saphenous vein^[1]^. However, the indications for the procedure are not precise. Surgeons generally prefer vessels with diameter ≥ 1.5 mm, supplying the viable myocardium^[1,4]^. It is performed especially when localized conditions prevent adequate distal flow^[2,6]^. Another situation that may require CE is the presence of a calcified or extremely hard plaque that ruptures with the suture or obstructs the flow, hindering the anastomosis^[2]^.

In this study, the largest number of patients from the two groups were men, without statistical difference. In the literature, CE is more frequent in male patients^[5,7]^. Although the female gender is a risk factor for mortality, CE is more associated with the male gender^[11]^. One possible cause is the shorter length and caliber of the arteries in women^[2]^.

CE can be programmed before arteriotomy, but it is generally an incidental procedure performed when the artery does not have conditions for an anastomosis with the graft, due to very intense atherosclerosis. Although the atherosclerotic involvement pattern can be predicted through coronary angiography, the decision to perform CE is rarely possible before the arteriotomy, being made in most cases during surgery^[2,6]^.

In 96.3% of cases, the endarterectomy was incidental, with only one patient undergoing the procedure on a scheduled basis. Tyszka et al.^[2]^ observed that, although the pattern of atherosclerotic involvement can be predicted by the analysis of the coronary angiography, the prediction of the need for endarterectomy is rarely possible. In the LAD artery, the endarterectomy may be required in case of coronary angiography showing a diameter < 1 mm with multiple severe stenosis (> 70%)^[12]^. However, as pointed out by Chi et al.^[13]^, the decision in relation to whether to perform CE or not is based on the results of preoperative imaging tests and intraoperative findings. The role of CE in CABG remains controversial, although it is often the only way to successfully treat some very calcified coronary arteries during surgery.

The endarterectomy has been performed more frequently in only one vessel, mainly the LAD artery (50% of cases), a fact already reported in other studies. The procedure in this vessel can be associated with increased morbidity and mortality, especially if the endarterectomy is performed by the closed technique^[7]^. In the literature, the right coronary artery (RCA) is the second vessel in which endarterectomy is more indicated, frequently using the closed technique^[7]^. We performed CE on branches of the left coronary artery in 70.37% of the cases, a result consistent with Tyszka et al.^[2]^. However, Chi et al.^[13]^ reported increased incidence of RCA in endarterectomy.

According to some authors, a greater number of vessels subjected to CE is associated with greater likelihood of mortality and complications, such as pneumonia, mediastinitis, perioperative bleeding, renal failure, atrial fibrillation, stroke, and, mainly, perioperative AMI, although these complications sometimes are associated with isolated CABG without CE^[1,4,10,14]^.

We performed CE in two vessels in two patients and both got discharged from the hospital. However, one of the patients had ischemic stroke on follow-up, after discharge. It should be emphasized that this patient was not using clopidogrel after hospital discharge. DAPT with aspirin and clopidogrel is routine management in patients undergoing CE, and 80% of patients followed this recommendation in this study. Wang et al.^[10]^ reported the association between CE and stroke in a meta-analysis, and the authors recommended the use of DAPT in order to prevent that complication.

The importance of coronary thrombosis prophylaxis in the clinical management of this group of patients, either through single or double antiplatelet therapy, has been demonstrated previously^[1,2,13]^. Russo et al.^[15]^ compared the effects of the single antiplatelet therapy to the DAPT (aspirin and clopidogrel) effects in patients undergoing CABG surgery associated with endarterectomy, and they did not find significant difference between the two groups at the end of the study. However, those authors recommend the administration of DAPT for a period of 12 months.

The incidence of perioperative AMI varies from 1.5% to 19%, according to the diagnostic criteria, and some studies showed that CE improved this incidence^[6,9,16]^. In this study, this incidence was within the limits mentioned in the literature, and it was similar in both groups.

We observed longer time of CPB and aortic clamp in patients undergoing CE, and these results are supported by other authors^[10,17]^. Soylu et al.^[9]^ also identified increase in aortic clamp time and CPB time in patients submitted to CE, which can lead to complications, such as renal hypoperfusion and coagulation disorders.

Among the CEG patients who died, two of them were submitted to LAD endarterectomy and one was submitted to endarterectomy of DGB. However, the death rate between the two groups showed no statistical difference (*P*=1.00). The death rate among CEG patients (8%) is consistent with the literature, ranging from 0% to 19%^[1,13]^.

The establishment of a CG, like in other studies, allowed the homogenization of the sample, providing data that suggest the benefit of CE in patients with complex coronary disease with low morbidity and mortality^[2,6]^.

Therefore, CE can be an important technique available to the cardiac surgeon. Recent studies indicate satisfactory patency rates with angiography and acceptable morbidity and mortality rates regardless of the vessel undergoing CE^[1]^. It is up to the surgeon to carefully check the indications for the procedure^[1,10]^.

In relation to early patency, 78.95% of the vessels submitted to CE were classified as patent, which is in accordance with the rates mentioned in the literature, ranging from 38% to 100%^[5,13]^. We consider our patency rate quite satisfactory and stimulating, considering that the arteries submitted to CE were of very poor quality. Many times, it would be impossible to perform grafts in these vessels without previous CE, and in other times, the grafts would have a high risk of occlusion because they would be performed on atheroma plaques. According to Soylu et al.^[16]^, the patency rate of the grafts in medium and long terms as well as the survival rate are better when open CE is combined with revascularization using the internal thoracic artery when compared to closed CE or open CE with the use of another graft. However, many studies do not specify which criteria were used to define graft failure (or non-patency) or its success^[5]^. Non-patency or anastomotic failure may be considered in the following cases: (I) total or 100% occlusion; (II) 75% stenosis or higher; (III) presence of tapering of the distal vessel by low flow^[4]^. In another study, non-patency was defined by total occlusion, stenosis above 90%, and presence of distal vessel tapering by low flow^[18]^.

Graft occlusion can be early or late. We consider early occlusion when it occurs in three to six months after surgery, usually as a result of thrombosis. On the other hand, late occlusion occurs usually due to the atherosclerotic process^[5]^.

As reported by Nishigawa et al.^[18]^, in a study with patients undergoing LAD endarterectomy, the patency rates in the angiographic study ranged from 83.3% to 99.5%, according to the graft used. The best results were achieved in patients who used LITA. We had statistically significant difference (*P*=0.0325) in relation to patency in the RCA and left coronary artery branches, which may be associated with the technique used or with RCA characteristics, which is frequently affected by important thickening and calcification^[2,7]^. Other causes of early graft occlusion include incomplete removal of atheromatous plaque, conditions of low cardiac output, and inadequate myocardial protection, as well as the type of graft used^[4,7,11]^. Despite the limitations of our study due to the small sample, it presented patency rates consistent with the literature, demonstrating the importance of the procedure on selected patients.

Regarding the limitations of the study, it is worth mentioning that it is a series of procedures performed by a single surgeon, who uses CE whenever necessary, the casuistry is small, and not all patients performed tests to control patency of the grafts. An examination to evaluate myocardial ischemia could also provide interesting information, but we do not perform it on a routine basis.

The use of the CE technique is still controversial, and the surgeons who defend it argue that many vessels would not be able to be grafted without it because of the great amount of calcium and the high risk of occlusion. The authors who oppose to its use justify their point of view by the risks of graft occlusion, longer surgical time, technical difficulties, and higher risk of mortality, and some believe that endarterectomy does not guarantee the distal flow because when we perform CE there is a risk of remaining plaques in the branches of the endarterectomized artery.

In order to clarify these questions, a prospective multicentric study of great statistical power might be necessary. In this study, patients with highly calcified arteries should be randomized to perform the isolated graft alone or CE, in order to later compare hard outcomes, such as stroke, AMI, and death, and the presence of residual ischemia in the endarterectomy region. However, conducting such a study is quite difficult, starting with the criteria of need for endarterectomy that are quite subjective, in addition to cost, training, and standardization aspects of endarterectomy techniques.

While we do not have definitive answers on the subject, the use of CE is dependent on the preference and experience of each surgeon. We believe that the technique should be used when well indicated. Indiscriminate use may lead to unnecessary procedures. However, in the present study, the technique was performed in only 8.4% of the grafts and it was restricted to situations in which the risk of anastomosis without CE was considered greater or even impossible. Generally, a few coronary grafts have early occlusion, already in the operating room, which can often be related to the quality of the treated coronary artery. Among the complicating factors, the presence of hard calcification has great relevance, and may contribute strongly to the early occlusion of the grafts^[19,20]^. It is noteworthy that there were cases in which the presence of calcification was so intense that it was not possible to transfix the calcium plate with the needles of polypropylene 7-0, 6-0, or up to 5-0, creating the need to remove the plate for anastomosis.

## CONCLUSION

In the present sample, the rate of early graft patency in patients submitted to CE was 78.9%. The comparison of the group submitted to CE with the CG showed that the former had a longer time of aortic clamping and longer time of CPB, however, there was no difference in the major endpoints such as stroke, AMI, or mortality.

**Table:** 

Authors’ roles & responsibilities	
MACC	Substantial contributions to the conception or design of the work; or the acquisition, analysis, or interpretation of data for the work; drafting the work or revising it critically for important intellectual content; agreement to be accountable for all aspects of the work in ensuring that questions related to the accuracy or integrity of any part of the work are appropriately investigated and resolved; final approval of the version to be published
ALB	Substantial contributions to the conception or design of the work; or the acquisition, analysis, or interpretation of data for the work; drafting the work or revising it critically for important intellectual content; final approval of the version to be published
JO	Substantial contributions to the conception or design of the work; or the acquisition, analysis, or interpretation of data for the work; drafting the work or revising it critically for important intellectual content; final approval of the version to be published
MS	Drafting the work or revising it critically for important intellectual content; agreement to be accountable for all aspects of the work in ensuring that questions related to the accuracy or integrity of any part of the work are appropriately investigated and resolved; final approval of the version to be published
ESR	Drafting the work or revising it critically for important intellectual content; agreement to be accountable for all aspects of the work in ensuring that questions related to the accuracy or integrity of any part of the work are appropriately investigated and resolved; final approval of the version to be published
RZG	Drafting the work or revising it critically for important intellectual content; agreement to be accountable for all aspects of the work in ensuring that questions related to the accuracy or integrity of any part of the work are appropriately investigated and resolved; final approval of the version to be published
